# Analyzing differentially expressed genes and pathways of Bex2-deficient mouse lung via RNA-Seq

**DOI:** 10.3906/biy-2104-4

**Published:** 2021-10-18

**Authors:** Noor BAHADAR, Hanif ULLAH, Salah ADLAT, Rajiv KUMAR SAH, May ZUN ZAW MYINT, Zin MAR OO, Fatoumata BINTA BAH, Farooq HAYEL NAGI, Hsu HTOO, Ahmad UD DIN, Xuechao FENG, Yaowu ZHENG

**Affiliations:** 1 Key Laboratory of Molecular Epigenetics, Institute of Genetics and Cytology, Northeast Normal University, Changchun, Jilin China; 2 School of medicine, Tsinghua University, Beijing China; 3 Drug Discovery Research Center, Southwest Medical University, Luzhou China

**Keywords:** CRISPR Cas9, knock-out mouse model, transcriptomic study, differentially expressed genes, KEGG pathwayactor

## Abstract

*Bex2* is well known for its role in the nervous system, and is associated with neurological disorders, but its role in the lung’s physiology is still not reported. To elucidate the functional role of *Bex2* in the lung, we generated a *Bex2* knock-out (KO) mouse model using the CRISPR-Cas9 technology and performed transcriptomic analysis. A total of 652 genes were identified as differentially expressed between *Bex2* ^-/-^ and *Bex2* ^+/+^ mice, out of which 500 were downregulated, while 152 were upregulated genes. Among these DEGs, *Ucp1, Myh6, Coxa7a1, Myl3, Ryr2, RNaset2b, Npy, Enob1, Krt5, Myl2, Hba-a2,* and* Nrob2 *are the most prominent genes. *Myl2*, was the most downregulated gene, followed by *Npy, Hba-a2, Rnaset2b, nr0b2, Klra8,* and *Ucp1*. *Tcte3, Eno1b, Zfp990,* and *Pcdha9* were the most upregulated DEGs. According to gene enrichment analysis, PPAR pathway, cardiac muscle contraction, and cytokine-cytokine receptor interaction were the most enriched pathways. Besides, the nuclear factor-κB signaling pathway and hematopoietic cell linage pathways were also enriched. Chronic obstructive pulmonary disease (COPD) is enriched among KEGG disease pathways. ﻿RT-qPCR assays confirmed the RNA-Seq results. This study opens a new window toward the biological functions of *Bex2* in different systems.

## 1. Introduction

Brain expressed X-linked (BEX), a gene family related to X chromosome, consists of BEX1 to BEX5 in humans, while in rodents, *Bex5 *is missing, instead, *Bex6* is found on chromosome 16 (Brown and Kay, 1999; Alvarez et al., 2005). All BEX genes consist of three exons, but only the third exon is coding for the protein (Fernandez et al., 2015).

hBEX2 gene was identified in 2002 in the embryonic cerebral cDNA library using high throughput sequencing technique (Yang et al., 2002), and *mBex2* in 1999 (Brown and Kay, 1999). A robust expression of *Bex2* has been observed in the mouse embryonic brain that plays a vital role in the development of the nervous system. BEX2 protein interacts with the hematopoietic transcription factor LIM domain only 2 (*LMO2*) and regulates transcriptional activity of an E-box sequence-binding complex that contains hBex2, LMO2, NSCL2 and LDB1 (Han et al., 2005). It has been reported that BEX2 is differentially expressed in breast tumors (Naderi et al., 2007). c-Jun and p65/RelA transcription factors targeting BEX2 are being phosphorylated in breast cancer cells. BEX2 activates the NF-kB pathway and thus inhibits ceramide 2 (C2) apoptosis of breast cancer cells (Naderi et al., 2007). An other group of researchers have identified that silencing of this gene promotes colorectal cancer metastasis through the Hedgehog signaling pathway (Tan et al., 2020).

*Bex2* is detected to be an oncogene in multiple types of cancers (Kazi et al., 2015). The gene plays a critical role in malignancies (Naderi, 2019). A genome-wide association study (GWAS) revealed that *Bex2* might act as a tumor inhibitor in glioma’s virulence (Foltz et al., 2006). *BEX2* plays a role in promoting human glioblastoma cells’ propagation via NF-kB signaling pathway (Meng et al., 2014). A very recent study explains the involvement of *Bex2* in the amelioration of allergic airway inflammation by adipose stem cell derived vesicles (Kim et al., 2020).

These results show that *Bex2* plays a critical role in multiple biological systems, especially in important oncogenic pathways. Gene expression analysis has found that *Bex2* is highly enriched in the lungs, but its function in the lungs has never been studied.

The transcriptomic analysis utilizes the second-generation DNA sequencing technology to measure the genome-wide gene expression levels (Costa et al., 2010). RNA-seq has been extensively used as a comprehensive approach to investigate various cell types and transcriptional patterns in transgenic mouse models (Adlat et al., 2020; Sah, et al., 2020). It is helpful to disclose novel genes and alternative splicing events (Trapnell, et al., 2012). Gene enrichment analysis can give a broad picture of genes involvement in pathways that predict its function and regulatory network (Kumar et al., 2016).

To further elucidate *Bex2* function in vivo, a mouse model was generated with *Bex2* global deletion (*Bex2* *^-/-^*) using the CRISPR-Cas9 system. The lung transcriptome analysis was carried out by performing RNA sequencing (RNA-seq). Although a *Bex2* knocked-out mouse has already been generated by other researchers (Ito et al., 2014), the transcriptomic analysis of lungs has not been studied to the best of our knowledge. The comparative analysis of differentially expressed genes (DEGs) between *Bex2* *^+/+^* and *Bex2* *^-/-^* indicates that several pathways are being enriched. This study provides important information on the potential biological functions of *Bex2*.

## 2. Materials and methods

### 2.1. Experimental animals

This study was approved by the Institutional Animal Care Committee and Animal Experimental Ethics Committee of Northeast Normal University. The care has been taken to minimize the discomfort of the experimental animals. All the recommendations for the use of laboratory animals of NIH (USA) were followed strictly. The mice were kept at the 12/12 light-dark cycle rotation in the pathogen-free environment with free access to food and water. The temperature of 21 °C was maintained along with 30%–60% humidity.

### 2.2. Plasmid construction for microinjection

The plasmid used, pX330-U6-Chimeric_BB-CBh-hSpCas9, was gifted from Feng Zhang (Cong et al., 2013), through Addgene (https://www.addgene.org/42230/). The single guided RNAs (sgRNAs) were designed (Table), using the Benchling database website (https://www.benchling.com), authenticated using BLAST tool of the NCBI (https://blast.ncbi.nlm.nih.gov/Blast.cgi) and annealed according to the previously described methods (Ran et al., 2013). Briefly, the forward and reverse sgRNAs were annealed in a buffer containing 150 mM NaCl and heated for 2 min at 94 °C and then gradually cooled down to 25 °C at the rate of 5 degrees per minute using BioRad thermocycler. The annealed oligos were then ligated to BbsI-digested and gel-purified plasmid vector pX330-U6-Chimeric-B-CBhSpCas9, using a ligation kit from Takara (Takara, Dalian, China). The *E. coli* strain DH5a was used for the transformation of the ligation product. Large prep of plasmid was obtained using alkaline lysis plasmid prep method. The final plasmids, psgRNA-*Bex2*-1 and psgRNA-*Bex2*-2, were confirmed by Sanger sequencing (https://www.genewiz.com.cn) using U6 promoter primers (Table).

**Table:** Genotyping primers and sgRNAs sequences.

Name	Sequence	Used for
sgRNA I	ctcttgtcttctaggagaaa	sgRNA target site I
sgRNA II	gactactacgtgcctagagg	sgRNA target site II
Bex2 F	ggatggatgggcgttagtcc	F primer genotyping
Bex2 R	gctcaggactcagggcataa	R primer genotyping
Bex2 qF	atcgtgcactacagatgggac	qRT-PCR F
Bex2 qR	tccaaagtggaacaaggcgtg	qRT-PCR R
U6 F	gaggcctatttcccatgatt	sgRNA ligation confirmation

### 2.3. Microinjection

The microinjection procedures were followed by the manual “Manipulating the Mouse Embryo; A laboratory Manual, 4th Ed.” by Cold Spring Harbor Laboratory. Ten 4-6-week-old C57BL/6J F1 females were superovulated by intraperitoneal injection with hormone PMGS (5 units in 100 uL saline) followed by the same PCG dose 40 hr later. The mating plug was checked the next day, and oocytes were isolated in M2 and cultured in M16. The mixes of the two plasmids (5 ng/uL each) were microinjected to the pronucleus of the fertilized oocytes, following the previously described methods (Wang et al., 2013).

### 2.4. Genotyping

The primer-BLAST tool was used to design* Bex2* specific primers located at the upstream and downstream of sgRNAs targeting sites (https://www.ncbi.nlm.nih.gov/tools/primer-blast/). All primer sequences are provided in Table. Finger biopsies of two-week-old pups were digested, and PCR genotyped. Briefly, the finger biopsies were digested in GNTK buffer including Proteinase K at 60 °C overnight, boiled for 10 min next morning, centrifuged for 5 min at 12,000 rpm for 5 min, and 0.8 uL was used as the template. The PCR specific conditions include denaturation at 95°C for 4 min, amplification at 94 °C, 56 °C each for 30 s, and 72 °C for 45 s, consist of 32 cycles followed by 10 min extension at 72 °C and then cooled down to 4 °C. The PCR amplified gene fragment was analyzed using 0.8% agarose gel electrophoresis. 

### 2.5. qRT-PCR

The total RNA was extracted from brain and lung tissues using RNAiso plus reagent (Takara, Dalian, China). The RNA concentration was measured using Nanodrop (ThermoFisher, USA) and 1 mg of total RNA was used to synthesize cDNA using Takara’s reverse transcription kit (Takara, Dalian, China). The RT-qPCR was carried out with SYBR green mix according to instructions (Takara, Dalian, China). The sequences of primers are listed in Table.

### 2.6. Extraction of RNA and library construction 

Total RNA was extracted from the lung of *Bex2* ^+/+^ and *Bex2* *^-/-^* mice, as mentioned above. The concentration, quality, and purity of RNA was confirmed on Nanodrop ND-2000 spectrophotometer (Thermo Fisher Scientific, USA), Agilent 2100 Bioanalyzer (Santa Clara, CA, USA), and through gel electrophoresis. One μg RNA for each sample was used for the RNA-seq library preparation. RNA-seq was accomplished on the BGISEQ platform with paired-end reads.

### 2.7. RNA-Seq data analysis

Low value reads, adaptor-only, and reads with more than one undisclosed base were distant using SOAPnuke (Cock et al., 2010) to acquire clean reads. Q20 (%), Q30 (%), and GC content (%) were analyzed. Reads were compiled into longer transcripts and aligned to reference genome (GCF_000001635.26_GRCm38.p6) using HISAT (Kim et al., 2015) and Bowtie2 tool (Langmead and Salzberg, 2012). The transcripts level was counted and presented as paired-end RNA-Seq FPKM (fragments per kilobase per million mapped reads) normalized reads. Differences in gene expression was recognized by annotation of two distinct libraries by applying Poisson distribution (Audic and Claverie, 1997) and expectation-maximization (RSEM) softwares (Li and Dewey, 2011). Following the normalization of the data, differentially expressed genes (DEGs) were analysed with |log2FC| ≥ +1 (upregulated), ≤ –1 (downregulated) and FDR <= 0.001 cut-off value. Kyoto Encyclopaedia of Genes and Genomes (KEGG) and Gene Ontology (GO) enrichment were executed for further functional analysis of distinctively expressed genes. According to DEGs, GO is functionally characterized as (i) biological process (BP), (ii) cellular component (CC), and (iii) molecular function (MF). According to their functional classes, GO functional enrichment was carried out using FDR ≤ 0.05 as the significant enrichment level. All the identified DEGs were aligned to the KEGG catalog for pathways analysis. Enrichment study of both GO and KEGG was executed using the phyper function in R software with a p-value < 0.05 (Kanehisa and Goto, 2000).

### 2.8. Statistical analysis

All the results are stated as means ± SEM (SEM). p-value < 0.05 (unpaired Student’s t-test) was considered statistically significant. The graphs were executed with GraphPad Prism 8 for Mac (GraphPad Software).

## 3. Results

### 3.1. Bex2^-/-^ mouse generation using CRISPR-Cas9 system

**Figure 1:**
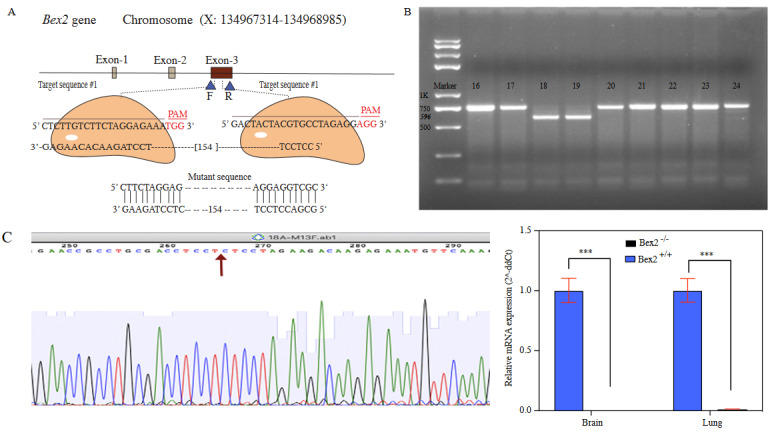
The strategy of Bex2 knock out and screening. (A) Graphical representation of the Bex2 gene and targeting sites. Exon-3 is targeted for deletion by designing two sgRNAs. Forward and reverse primers flanking the two sgRNAs sites were intended for genotyping. (B) Genotyping of the pups by PCR. Two pups (#18 and #19) showed knocked-out bands (Bex2-/-). (C) Sanger sequencing chromatogram of the deletion site. Arrow indicates the deletion joint. (D) qRT-PCR confirmation of Bex2 mRNA loss in brain and lungs.

The *Bex2* is a small gene and consists of only 1672 bps. Only the third exon contains the entire coding sequence (Figure 1A). To knock out the gene, the sgRNA1 was designed in front of the start codon, while the sgRNA2 (Table) was targeted downstream of the start codon but before stop codon (Figure 1A). To screen mutations, primer pair was designed flanking sgRNA targets with 752 bps of PCR product (Table).

Nine pups were obtained, numbered from 16 to 24 (Figure 1B). Fingers biopsies were used for genotyping, following the standard protocols, using forward and reverse primers (Table). The PCR product was subcloned into pMD18 plasmid (Takara, China) and sequenced. The sequencing results assured the correct deletion between two target sites (Figure 1C). Pups number 18 and 19 were found to be transgenic. These two mice were mated with wild-type C57BL/6 to confirm germline transmission. Downstream progenies were used for experimental analysis.

### 3.2. Confirmation of Bex2 expression level in lung and brain by qPCR

We performed quantitative real-time PCR (qPCR) to identify the distinctively expressed genes in the lung and brain between wild-type and knock-out mice. The qPCR primers are provided (Table). The mRNA levels in both lungs and brain were compared between *Bex2* ^-/- ^and *Bex2* *^+/+^*. The relative mRNA expression results showed loss of the *Bex2* (Figure 1D).

### 3.3. An overview of RNA-Seq libraries

The cDNA libraries were generated from lung mRNAs of Bex2-/- and Bex2+/+ mice (n = 3). BGISEQ platform was used for RNA-seq. The average yield was 6.77G for each sample. The average alignment ratio of each sample to reference sequence (Mus musculus genome reference GCF_000001635.26_GRCm38.p6) was 95.67%. The average alignment of gene set was 73.74% with a total of 17,244 genes detected. Number of genes in each FPKM level (FPKM <= 1, FPKM 1–10, FPKM> = 10) is listed in Table S1. In the first category, FPKM <=1, 4605 genes were expressed in Bex2-/- while 4379 genes in Bex2+/+. In FPKM 1–10 category, 6281 genes were identified in Bex2-/- and 6245 genes in Bex2+/+. In the category FPKM >=10, 6358 genes were found in Bex2-/- and 6620 genes in Bex2+/+.

### 3.4. Differentially expressed genes identification in Bex2^-/-^ ****lung

**Figure 2:**
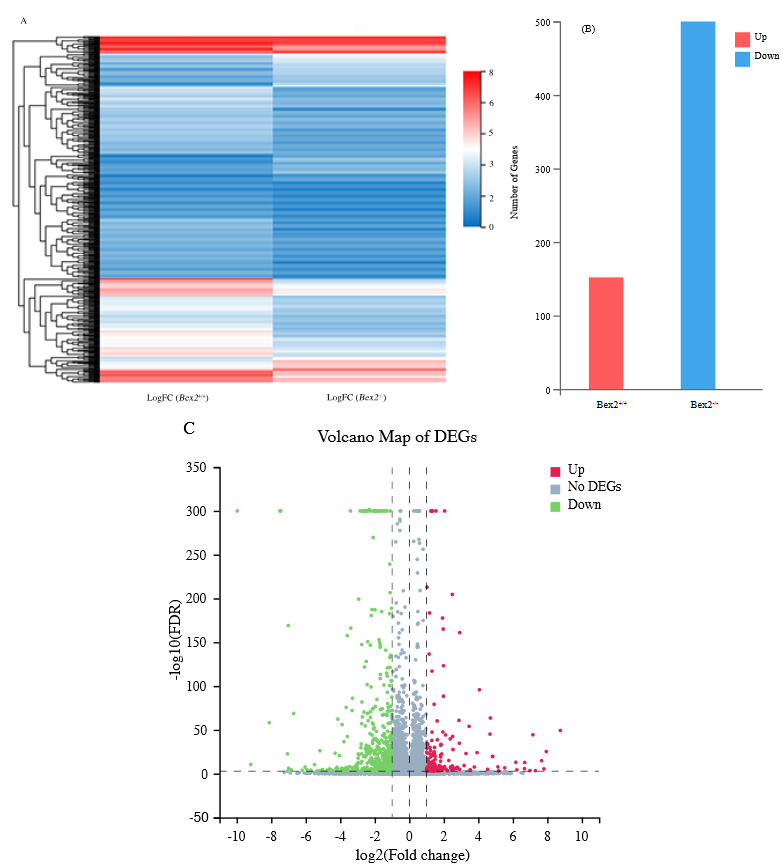
An outline of the DEGs between Bex2-/- and Bex2+/+. (A) Heatmap presentation of the DEGs. The color gauge from 0 to 8 indicates the gene expression level (log2FC). (B) Statistics of the upregulated and downregulated genes. (C) Volcano plot representing the DEGs. Fold change difference after conversion to log2 along X-axis and, significance value after conversion to -log10 along Y-axis. Red dots represent DEGs upregulated, blue dots represent DEGs downregulated, and grey dots represent non-DEGs.

Hierarchical clustering was accomplished for the genes expressed only in *Bex2* *^-/-^* according to the difference in fold change values (log_2_FC). To see the gene expression and regulation (up/down) analysis, we performed a pairwise comparison between *Bex2* ^-/-^ and *Bex2* ^+/+^. A heatmap of the gene expression is provided (Figure 2A). We identified a total of 652 unigenes that were differentially expressed (DEG) (152 upregulated and 500 downregulated) in the lungs between *Bex2* ^-/-^ and *Bex2* ^+/+^ (Figure 2B). According to each sample’s gene expression level, the significant DEGs detected were statistically plotted, and a volcano graph has been devised (Figure 2C).

### 3.5. DEG functional enrichment analysis

**Figure 3:**
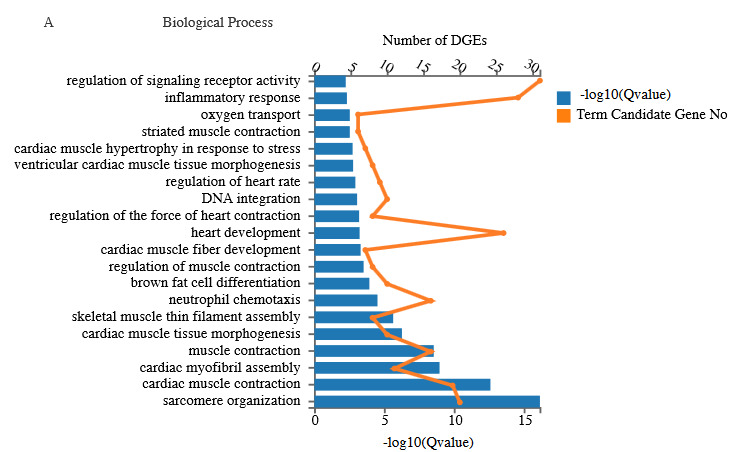

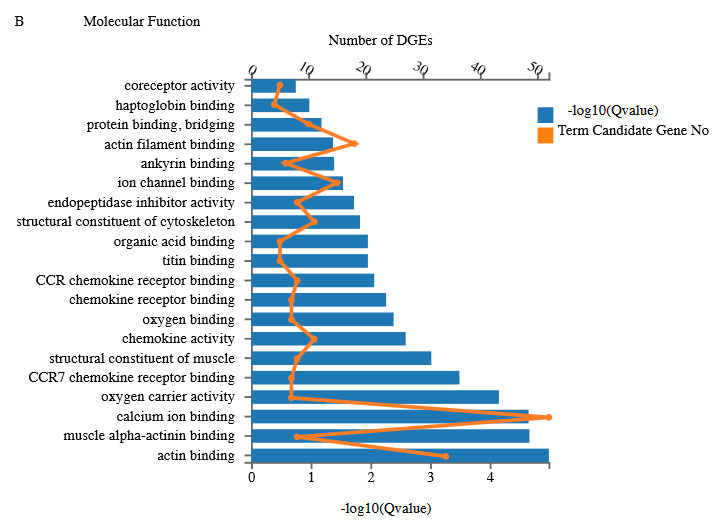

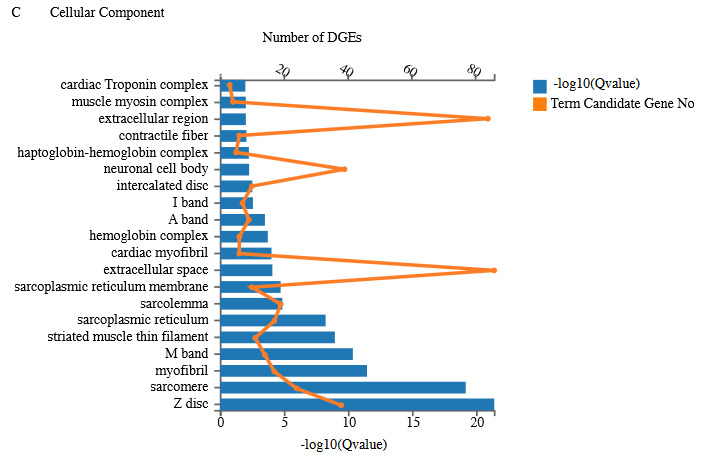
Gene Ontology (GO) analysis between Bex2-/- and Bex2+/+ showing the different enriched ontologies of DEGs. (A) Biological process enrichment. (B) Molecular function enrichment. (C) Cellular component enrichment.

Gene ontologies offer a systematic language for genes and their products in three terms: molecular function (MF), biological process (BP) and cellular component (CC) (Ashburner et al., 2000). Significant DEGs in the BP category contains regulation of the signaling receptor activity, inflammatory response and oxygen transport (Figure 3A). For the MF category, coreceptor activity, haptoglobin binding and protein binding bridging were prominent (Figure 3B). In the CC category, cardiac troponin complex, contractile fiber muscle myosin complex were the leading terms (Figure 3C).

**Figure 4:**
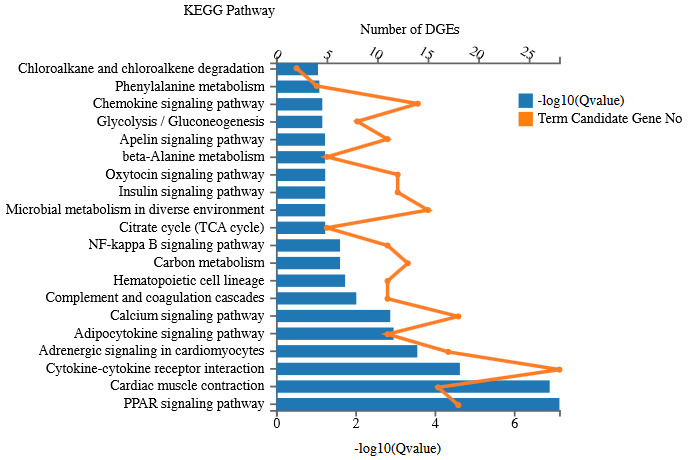
KEGG pathway enrichment of DEGs.

Similarly, all the DEGs (652) were assigned for KEGG pathway analysis to see DEGs’ contribution in different pathways. Enrichment analysis confirmed a total of 217 KEGG pathways. The most enriched pathways are PPAR signaling pathway, cytokine-cytokine receptor interaction, adrenergic signaling in cardiomyocytes, adipocytokine signaling pathway, complement and coagulation cascades, calcium signaling pathway, hematopoietic cell lineage, carbon metabolism, NF-kB signaling pathway, and many more (Figure 4).

### 3.6. KEGG analysis of disease-associated pathways

**Figure 5:**
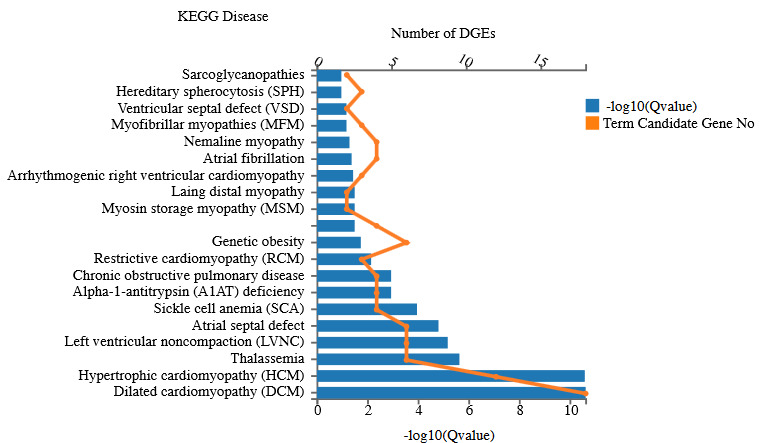
KEGG disease analysis.

All the DEGs were annotated to KEGG disease enrichment (Figure 5). The most significant pathways identified are dilated cardiomyopathy (DCM), hypertrophic cardiomyopathy (HCM), thalassemia, left ventricular noncompaction (LVNC), arterial septal defect, sickle cell anemia (SCA), alpha-1-antitrypsin (A1AT) deficiency, chronic obstructive pulmonary disease (COPD), etc. Among these, the COPD is related to lung physiology. Members of *Serpina1* are involved in both A1AT and COPD.

### 3.7. Genes that encoding transcription factor proteins

**Figure 6:**
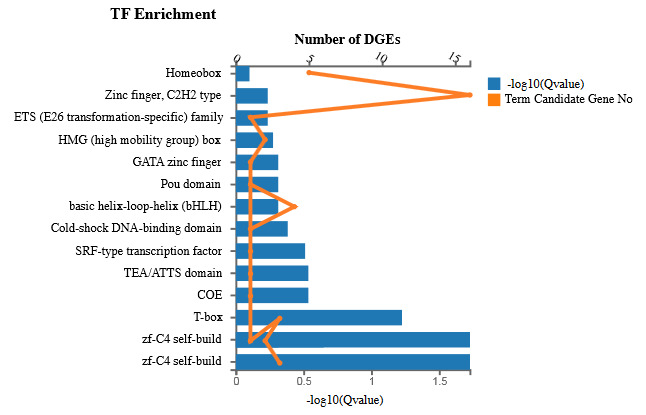
Expression profiling of transcription factors.

Transcription factors (TFs) are the essential regulatory proteins that play roles in multiple biological processes. A certain number of TFs were identified in this study. The most enriched TFs are zf-C4 self-build, T-box, COE, TEA/ATTS domain, SRF transcription factor, cold shock DNA binding domain, and others (Figure 6).

### 3.8. DEGs validation by qPCR

**Figure 7:**
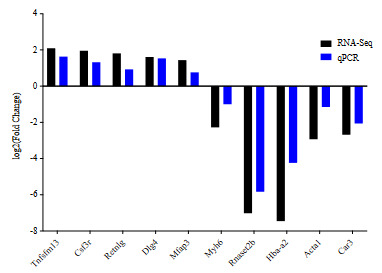
Validation of RNA-seq results by qPCR. Selected DEGs from RNA-seq are confirmed. Fold change expression level along X-axis while Y-axis representing gene name.

To confirm the results of RNA-seq, ten genes (five upregulated and five downregulated) were measured for their relative expression level by qPCR. These genes were selected based on their important roles in lung development and function including *Tnfsfm13, Csf3r, Retnlg, Dlg4, Mfap3, Myh6, Rnaset2b, Hba-a2,* *Acta1* and *Car3*. RNA–seq results are consistent with RT-qPCR results (Figure 7).

## 4. Discussion

*BEX2* expression was favorably found in embryonic brain, which has a vital role in the nervous system development and related neurological disorders (Han et al., 2005). The gene is predominantly expressed in the brain, but the significant expression is found in other tissues, such as the embryonic liver, adult placenta, and lungs. Although the *Bex2* KO mouse model has been generated (Ito et al., 2014), using a gene targeting strategy, the lung’s transcriptomic study has not been reported yet. In this study, we used CRISPR-Cas9 technology to generate a global knock-out mouse model of the *Bex2* gene. The RT-qPCR analysis shown that the *Bex2* is highly expressed in brain and lungs. The relative mRNA expression level analysis by RT-qPCR of the knocked-out mouse brain and lung indicated that the gene is successfully deleted. We performed RNA-seq analysis from the lung tissue to investigate the genes and pathways under the control of *Bex2* *^-/-^*. Surprisingly, most of the enrichment is related to the cardiac system. Similar results were obtained by other reserachers also (Yee et al., 2018).

BEX2 is differentially expressed in breast tumors (Naderi et al., 2007), acute myeloid leukemia, and an increased expression was found in the MLL subtype (Rohrs et al., 2009). Lungs are not only the respiratory organs, but there are pieces of evidence that they also play a role in immunity (Lefrançais et al., 2017). We found that most of the KEGG pathways enriched in the immune system. PPAR signaling pathway, and cardiac muscle contraction, are the most significantly enriched pathways. Similarly, we found a prominent contribution of DEGs in complement and coagulation cascades and hematopoietic cell lineage pathways. We found that *Tnfsfm13*, a member of TNFSF, is upregulated (DEG, log_2_ = 2.09), indicates its role in synergistic effect with *Bex2*. *Retnlg* (DEG, log_2_ = 1.81) is most likely expressed in nonadipose tissues (Lefrançais et al., 2017), indicates its possible role in the inflammation of lungs. *CSF3*, otherwise identified as G-*CSF*, exists within the primary 17q12-21 asthma predisposition and exacerbation locus (Bisgaard et al., 2009). *Csf3r* found in our upregulated DEGs (log_2_ = 1.96) following the results of other researchers (Wang et al., 2019), may play a role in asthma. Similarly, the downregulated genes are comparable to the results of the other researchers. For instance, *Myh6* among downregulated genes (log_2_ = -2.27) is following the results of other researchers (Yee et al., 2018), where they found that neonatal hyperoxia reduced the gene expression involved in contractile function (like *Myl4*, *Myl7*, *Myh6*, *Myh7*) etc. A detailed study is needed to cross-check the synergism among these gene functions.

BEX2 has been shown that it activates the NF-kB signaling pathway in breast cancer cells (Naderi et al., 2007), promotes the human glioblastoma cells proliferation via the NF-κB signaling pathway (Meng et al., 2014). In brain tumors, BEX2 enhances cell moment and invasion in oligodendroglioma and glioblastoma cells. Besides, the expression of BEX2 protects glioma cells against apoptosis mediated via the JNK pathway and is essential for glioma cell proliferation through the NF-κB p65 (Naderi, 2019). In this study, NF-κB is one of the prominent enriched pathway. The most enriched gene among them is *Tnf, *which plays a central role in the development of the immune system. Several genes from mTOR Pathway were enriched including *Prkaa2* log_2_ –1.08, *Sos2* log_2_ –1.10, *Tnf* 1.64 and *Rragd* log_2_ –1.84. Similarly, *Bcl2a1d*, from NF-kB pathway is related to apoptotic process (Cartagena et al., 2013). *Bdkrb2*, log_2_= 1.62, has a functional role related to reactive oxygen species (Perhal et al., 2019).

 A more detailed study is needed to determine the synergism of *Tnf* and *Bex2*. A recent study explains the role of BEX2 gene in allergic airway inflammation (Kim et al., 2020).

In cell lines of leukemia, BEX2 was found to be expressed in MLLmu AML (Quentmeier et al., 2005). DEGs that enriched hematopoietic cell lineage pathway indicate that several genes e.g, *Gm13305* log_2_: 4.82, *Cd19* log_2_: 1.02, *Cd22* log_2_: 1.20, *Csf3r* log_2_: 1.96, *Il11a2* log_2_: 3.92, *Il1r2* log_2_: 2.04, etc. were related to immune system. Besides, several DEGs identified were involved in the endocrine system and metabolic pathways. A more detailed study is required to use specific markers to validate the results of this bioinformatics analysis.

## 5. Conclusion

In this study, a mouse model of *Bex2* global knock-out has been generated using the CRISPR Cas9 system. The research of genes and pathways under *Bex2* regulation has identified many potentially important roles of this gene. We identified that several pathways are enriched related to the immune system. Besides the functions in respiration, the lungs also play roles in the immune system and maybe a possible site for platelets’ biogenesis. Moreover, several metabolic pathways are also identified. Several cardiac-related genes are found in DEGs. A detailed study is needed to determine the synergism between *Bex2* and the most prominent DEGs.

## Funding

This research was funded by National Natural Science Foundation of China, grant number 31301189 and 81270953 and Natural Science Foundation of Jilin Province, grant number 20200201127JC 20160101344JC, Science and Technology Project of Jilin Provincial Education, grant number JJKH20180023KJ. 
